# Extranodal Marginal Zone B-cell Lymphoma Presenting as a Painless Buccal Mass in the Masticator Space

**DOI:** 10.14740/jmc5203

**Published:** 2026-03-04

**Authors:** Li Lin, Jiun-Sheng Lin

**Affiliations:** a Department of Oral and Maxillofacial Surgery, MacKay Memorial Hospital, Taipei, Taiwan, Republic of China; b Institute of Oral Biology, School of Dentistry, National Yang Ming Chiao Tung University, Taipei, Taiwan, Republic of China

**Keywords:** Marginal zone lymphoma, MALT lymphoma, Masticator space, Buccal mass, Head and neck lymphoma, Radiotherapy, Rituximab

## Abstract

Extranodal marginal zone B-cell lymphoma (EMZL, mucosa-associated lymphoid tissue type) is an indolent non-Hodgkin lymphoma that only rarely arises in deep facial spaces. Primary masticator-space involvement is particularly uncommon and can mimic benign buccal soft-tissue lesions. We report a 67-year-old woman with a 4-year history of a painless, slowly enlarging left cheek mass. Examination showed a soft, mobile 3-cm buccal swelling with normal overlying skin and no intraoral lesion. Contrast-enhanced computed tomography demonstrated an infiltrative soft-tissue mass measuring 3.1 × 1.5 × 3.5 cm, centered in the left masticator space, with effacement of fat planes between the masseter, pterygoid, and temporalis muscles. Intraoral incisional biopsy revealed a dense infiltrate of small-to-medium CD20-positive, Bcl-2-positive B cells, with a CD5, CD10, CD23, Bcl-6-negative immunophenotype and follicular colonization on CD21 staining, consistent with EMZL. A multidisciplinary tumor board recommended involved-site radiotherapy to the masticator space combined with rituximab-based immunotherapy. This case represents a rare example of primary EMZL arising in the masticator space, which highlights the need to include lymphoma in the differential diagnosis of persistent head and neck masses, to obtain tissue diagnosis for infiltrative head and neck lesions and to coordinate organ-preserving treatment through multidisciplinary care.

## Introduction

Extranodal marginal zone B-cell lymphoma (EMZL) accounts for roughly 7–8% of B-cell lymphomas and typically presents in older adults as a slow-growing mass at mucosal or glandular sites [1, 2]. Common primary locations include the stomach, ocular adnexa, salivary glands, lung, thyroid, and skin [1, 3]. In the head and neck, most EMZL arises in salivary glands or ocular adnexa; primary disease centered in the bucco-masseteric or masticator spaces is rare and may be easily misattributed to benign conditions because constitutional symptoms are often absent [4, 5]. Early recognition matters: localized, non-gastric EMZL is highly radiosensitive, and organ-preserving therapy yields durable control in most patients [2, 6, 7]. We report an unusual masticator-space EMZL presenting as a painless buccal mass and discuss practical points in diagnosis, staging, and management for maxillofacial clinicians.

In daily practice, a painless, slow-growing buccal swelling is more often attributed to benign lesions such as lipoma, venous malformation rather than to lymphoma, particularly when the overlying mucosa is normal and dentition is intact. Deep lesions in the bucco-masseteric or masticator spaces may also be misinterpreted as odontogenic infection or temporomandibular joint pathology. Because primary lymphoma of the masticator space is exceptionally uncommon and frequently presents without B symptoms, a simple excision or even observation can delay diagnosis and treatment. This report describes a rare case of primary EMZL arising in the masticator space, emphasizes the imaging and pathologic clues that distinguish it from more common buccal masses, and reviews current evidence to guide staging and management.

## Case Report

A 67-year-old woman presented with a 4-year history of a slowly enlarging, painless swelling in the left cheek. The swelling was steady and non-bothersome; there was no tenderness, redness, trismus, dysphagia, weight loss, fever, or night sweats. She had no known systemic diseases. Past operations included excision of a left facial neurilemoma and removal of a neck epidermal inclusion cyst 5 years earlier. She took no regular medications and reported no tobacco or alcohol use.

**Figure 1:**
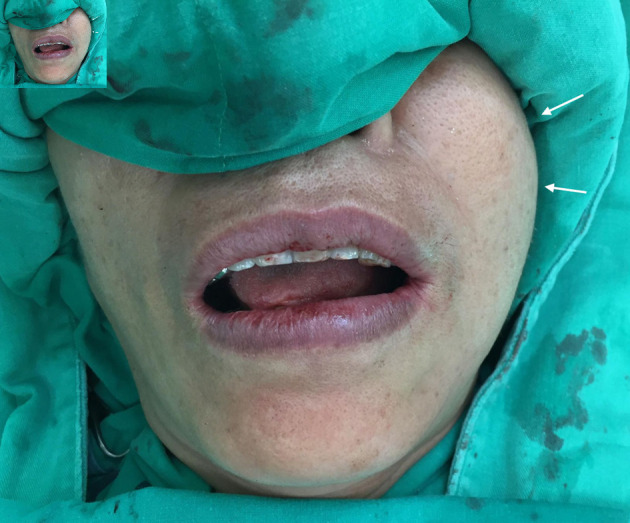
Physical examination revealed a soft, mobile, subcutaneous mass (about 3 cm) in the left buccal region (arrows).

On examination, there was a soft, mobile, subcutaneous mass in the left buccal region measuring approximately 3 cm. Overlying skin was intact and normal in color and temperature; cranial nerve function was preserved. Intraoral inspection showed no mucosal lesions, swelling of Stensen’s duct, or dental pathology. No cervical lymphadenopathy was palpable (Fig. 1).

**Figure 2:**
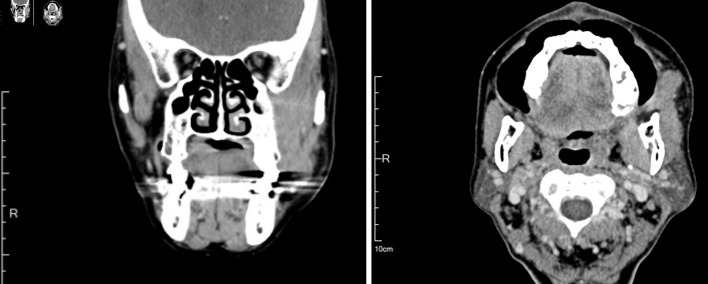
Contrast-enhanced computed tomography revealed an infiltrative soft-tissue mass measuring 3.1 × 1.5 × 3.5 cm, centered in the masticator space with effacement of fat planes between the masseter, pterygoid, and temporalis muscles.

**Figure 3:**
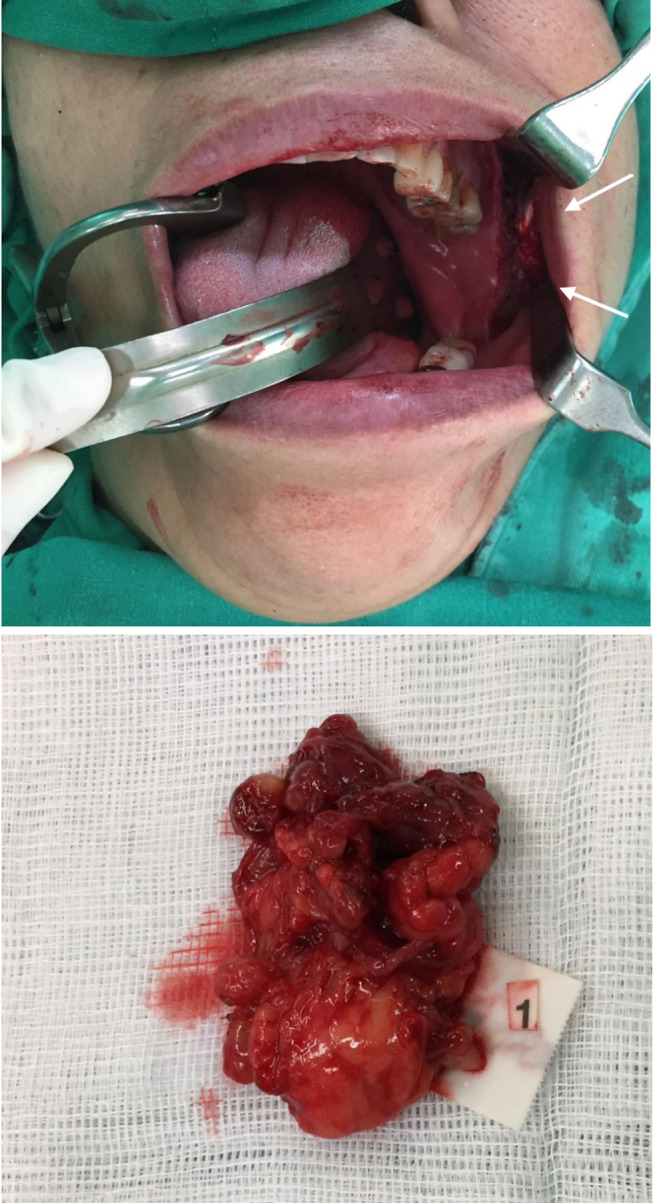
An intraoral incisional biopsy under general anesthesia with obtained specimen (arrows). The intraoral approach allowed direct access to the bucco-masseteric space while avoiding external scarring.

Contrast-enhanced computed tomography (CT) of the face revealed an infiltrative soft-tissue mass measuring 3.1 × 1.5 × 3.5 cm, centered in the left masticator space, with effacement of fat planes between the masseter, pterygoid, and temporalis muscles; there was no cortical erosion (Fig. 2). Given the deep-space location and infiltrative appearance, an intraoral incisional biopsy was performed under general anesthesia (Fig. 3).

**Figure 4:**
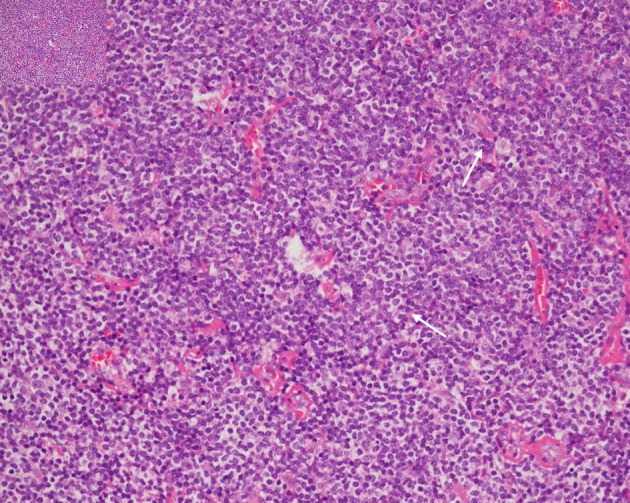
H&E stain (× 40) demonstrating a diffuse, vaguely nodular infiltrate, expanding the soft tissue and disrupting normal architectural boundaries. Note the presence of mild plasmacytic differentiation (arrows). H&E: hematoxylin and eosin.

**Figure 5:**
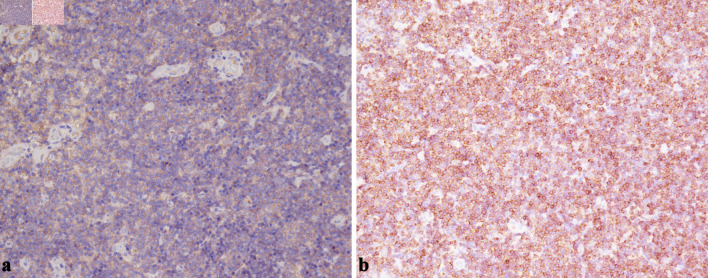
(a) Representative immunohistochemistry demonstrating diffuse CD20 positivity in the neoplastic B cells within the masticator-space lesion (× 200). (b) Bcl-2 staining highlights strong cytoplasmic expression in the small B cells, supporting a diagnosis of EMZL rather than reactive hyperplasia (× 200). EMZL: extranodal marginal zone B-cell lymphoma.

**Figure 6:**
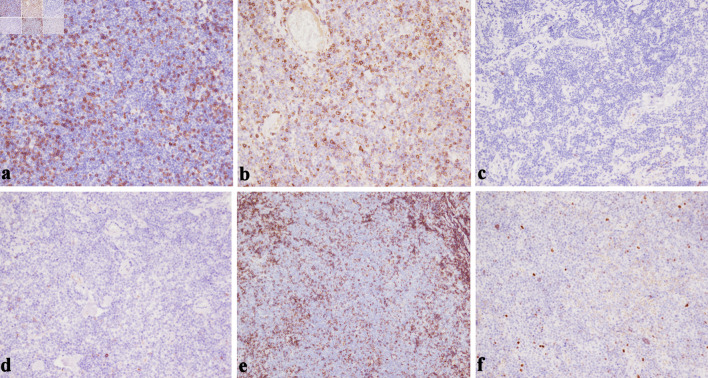
(a) CD3 negativity in the tumor cell population confirms that the predominant infiltrate is of B-cell rather than T-cell lineage (× 200). (b) Lack of CD5 expression helps to exclude mantle cell lymphoma and most chronic lymphocytic leukemia/small lymphocytic lymphoma (× 200). (c) The neoplastic lymphoid infiltrate is negative for CD10, supporting a marginal zone origin and helping exclude follicular lymphoma (× 200). (d) The neoplastic lymphoid population is negative for CD23 expression, helping distinguish this case from small lymphocytic lymphoma (× 200). (e) CD43 negativity in the neoplastic B cells is consistent with an EMZL phenotype and argues against other small B-cell lymphomas (× 200). (f) Bcl-6 negativity in the neoplastic lymphoid cells (× 200). This lack of Bcl-6 expression supports a post-germinal center origin and helps exclude follicular lymphoma. EMZL: extranodal marginal zone B-cell lymphoma.

**Figure 7:**
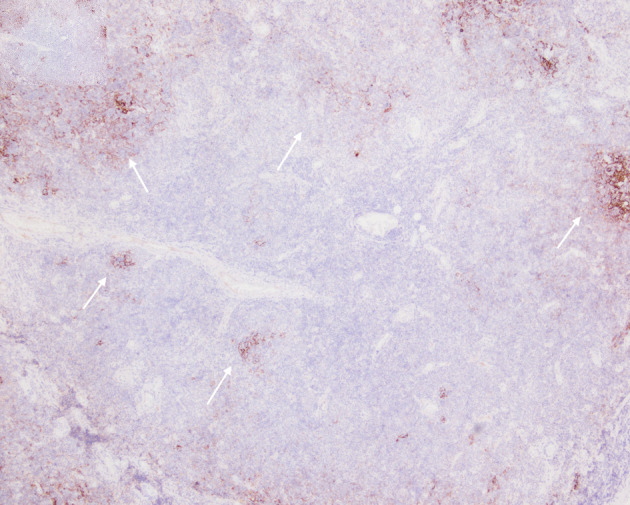
CD21 outlining disrupted follicular dendritic cell meshworks consistent with follicular colonization (arrows).

Upon histopathologic examination, hematoxylin and eosin (H&E) stain showed a dense, monotonous infiltrate of small-to-medium lymphoid cells with irregular nuclear contours, clumped chromatin, inconspicuous nucleoli, and abundant pale cytoplasm, admixed with residual reactive follicles and accompanied by mild plasmacytic differentiation (Fig. 4). Immunohistochemistry demonstrated strong CD20 and Bcl-2 positivity in tumor cells (Fig. 5) and negativity for CD3, CD5, CD10, CD23, CD43, and Bcl-6 (Fig. 6a–f). CD21 outlined disrupted follicular dendritic cell meshworks, consistent with follicular colonization (Fig. 7). The findings were diagnostic of extranodal marginal zone B-cell lymphoma (mucosa-associated lymphoid tissue (MALT) type). The case was reviewed at a multidisciplinary tumor board. In view of the localized presentation and radiosensitive biology of EMZL, the consensus recommendation was involved-site radiotherapy (ISRT) to the masticator space with anti-CD20 immunotherapy (rituximab). Despite counseling about the favorable risk-benefit profile, the patient did not return to initiate therapy and was lost to follow-up.

## Discussion

The multi-year, painless enlargement of a buccal mass with preserved function aligns with the indolent biology of EMZL and illustrates why lymphoma may be overlooked in deep facial spaces [2, 4]. In the buccal region, the differential diagnosis for a soft, mobile mass spans lipoma, benign peripheral nerve sheath tumor, venous malformation, accessory parotid or minor salivary lesions, nodular fasciitis, metastatic lymphadenopathy, and sarcomas [4, 8]. A pragmatic red flag is progressive enlargement with cross-sectional imaging that depicts an infiltrative soft-tissue process and blurring of normal fat planes but little necrosis or calcification; frank bone erosion is uncommon at presentation in indolent lymphomas [2, 8]. In our patient, the CT finding of fat-plane effacement between the masseter, pterygoid, and temporalis muscles—without cortical breach—favored deep-space pathology and appropriately prompted biopsy rather than excision of a presumed lipoma. The 4-year history of a persistent and slowly progressive swelling in our patient illustrates how easily such indolent lymphomas can be overlooked and mistaken for benign soft tissue lesions in the absence of pain, mucosal change and dental pathology.

Diagnosis rests on tissue. EMZL typically shows small-to-medium, centrocyte-like/monocytoid B cells, with characteristic follicular colonization—well highlighted by CD21/CD23 outlining of residual follicular dendritic networks [1, 2]. The immunophenotype (CD20^+^/Bcl-2^+^ and CD5/CD10/CD23/Bcl-6 negative) is instrumental in excluding mantle cell lymphoma, follicular lymphoma, and small lymphocytic lymphoma [1–3]. Our specimen’s morphology and profile were classic for EMZL with follicular colonization. Cytogenetic events that converge on NF-κB activation—such as BIRC3-MALT1, IGH-MALT1, and BCL10-IGH translocations—are documented across sites; although not required for diagnosis, they explain aspects of pathogenesis and, in gastric disease, may bear on antibiotic responsiveness [1, 2]. In our case, plasmacytic differentiation was mild and did not obscure the underlying small B-cell infiltrate, but its presence further supported an EMZL phenotype rather than a purely nodal or follicular lymphoma.

Staging of head-and-neck EMZL should be thorough yet pragmatic. Although many patients present with limited-stage disease, non-gastric sites exhibit a meaningful rate of mucosal multifocality or systemic spread [1, 2]. Baseline workup generally includes history/physical examination; complete blood count, chemistry, and lactate dehydrogenase (LDH); hepatitis B screening before rituximab; and contrast-enhanced CT of neck/chest/abdomen/pelvis [2, 6]. Bone marrow evaluation is considered when results would change management. Positron emission tomography (PET) has variable sensitivity in EMZL—detection tends to be higher in non-gastric sites than in gastric lymphoma—but a negative PET does not exclude disease [2]. Risk can be summarized with the mucosa-associated lymphoid tissue–international prognostic index (MALT-IPI) (age ≥ 70, stage III/IV, elevated LDH), which stratifies event-free survival; most localized head-and-neck cases fall into lower-risk groups [1, 6]. Primary lymphoma centered in the masticator space is rare and has been reported mainly as isolated case reports and small series. Published cases include extranodal marginal zone lymphoma and other histologic subtypes, typically presenting as cheek, parotid, temporal-region swellings that may be mistaken for benign or inflammatory lesions [9–12]. To our knowledge, the present case therefore represents the second reported instance of primary EMZL arising in the masticator space.

Management of localized, non-gastric EMZL is organ-preserving and highly effective. For head-and-neck sites, ISRT delivered at modest doses (approximately 24–30 Gy) achieves high complete response rates and durable local control with low toxicity [6, 7, 13]. Across series involving lacrimal, salivary, and thyroid presentations, local control commonly exceeds 90% with contemporary planning [6, 7]. Rituximab may be used alone for patients unfit for radiation or when systemic control is needed. Combined regimens (e.g., rituximab with alkylators) are appropriate for disseminated or symptomatic EMZL, with randomized data supporting the addition of rituximab to chlorambucil to improve event-free survival [6]. Chemo-free options such as rituximab–lenalidomide show activity in indolent lymphomas, including EMZL [6]. Our tumor board’s recommendation of ISRT plus rituximab aimed to maximize local control while addressing the possibility of microscopic multifocality—an approach consistent with modern practice for localized non-gastric EMZL [2, 6, 7]. Beyond conventional chemoimmunotherapy and radiotherapy, recent series and reviews have underscored the biological heterogeneity of marginal zone lymphoma and the expanding therapeutic armamentarium, including Bruton tyrosine kinase inhibitors and lenalidomide-based combinations, which may be considered in relapsed, refractory, or disseminated disease [14–17].

Follow-up requires intention. Head-and-neck EMZL usually behaves indolently but can relapse locally or at distant mucosal sites years later [4, 18]. Transformation to diffuse large B-cell lymphoma is uncommon but clinically important; it should be suspected in the setting of accelerated growth, new B symptoms, or rising LDH, prompting biopsy of the most suspicious or fluorodeoxyglucose (FDG)-avid site [2, 6]. In our case, loss to follow-up—despite a favorable risk-benefit profile—highlights a practical barrier to cure. Providing clear written instructions, scheduling treatment steps (e.g., radiotherapy simulation) before discharge, offering multiple contact pathways, and engaging nurse navigators can reduce attrition and help translate EMZL’s favorable biology into durable benefit. For this discussion, relevant literature was identified by searching PubMed for the terms “masticator space lymphoma,” “marginal zone lymphoma,” and “MALT lymphoma” and by screening the reference lists of retrieved articles.

For maxillofacial clinicians, several lessons emerge. Persistent, painless buccal or masticator-space masses merit cross-sectional imaging; infiltrative margins with fat-plane effacement should prompt biopsy rather than *en bloc* excision of a presumed benign lesion. Intraoral biopsy routes can minimize morbidity while providing sufficient tissue for advanced hematopathologic evaluation. Once EMZL is confirmed, staging, risk assessment, and organ-preserving therapy—often ISRT with or without rituximab—offer excellent outcomes [1, 7, 13]. Finally, building structured follow-up into the plan from the outset is essential, particularly for patients who feel well and may underestimate the need for treatment. The present case therefore reinforces several practical learning points: long-standing buccal swellings with infiltrative imaging features should be considered possible lymphomas; a tissue diagnosis should be obtained rather than proceeding directly to excision; and time should be invested in structured counseling and coordinated appointments so that patients understand the rationale, safety, and importance of completing organ-preserving therapy.

### Conclusions

A long-standing, painless buccal mass with infiltrative features on imaging should raise suspicion for lymphoma in addition to common benign entities. Classic morphology with a CD20^+^/Bcl-2^+^, CD5/CD10/CD23-negative immunophenotype and follicular colonization supports EMZL (MALT type). For localized head-and-neck disease, ISRT—with or without rituximab—achieves excellent control with organ preservation. Early multidisciplinary planning and proactive follow-up are key to realizing this benefit. Taken together, this rare presentation of primary EMZL in the bucco-masseteric space underscores that even indolent lymphomas can masquerade as harmless soft-tissue enlargements. Systematic assessment of history, imaging, and histopathology, followed by evidence-based, organ-sparing therapy and proactive follow-up, can convert EMZL’s favorable biology into durable functional and oncologic outcomes.

### Learning points

A long-standing, painless buccal or cheek swelling with normal mucosa and no clear dental cause should not be dismissed as “just a lump.” It should prompt cross-sectional imaging (CT or MRI) and consideration of lymphoma, especially if the scan shows an ill-defined, infiltrative lesion in the masticator space rather than a neat, well-circumscribed mass.

EMZL cannot be reliably distinguished from reactive lymphoid hyperplasia, other small B-cell lymphomas, or non-lymphoid tumors by clinical exam alone. A proper incisional biopsy from the deep part of the lesion, combined with a full immunohistochemical workup (e.g., CD20, Bcl-2, CD3, CD5, CD10, CD23, Bcl-6, CD21, ± light-chain studies) is essential for an accurate diagnosis.

Localized head-and-neck EMZL, including rare masticator-space cases, is usually highly radiosensitive and can often be managed successfully with ISRT plus anti-CD20-based systemic therapy in a multidisciplinary setting, allowing good disease control with preservation of function and appearance.

Because many patients feel well and symptoms are mild, they may underestimate the seriousness of the diagnosis. Clear explanation, simple written information, and proactive scheduling of treatment and follow-up are crucial to prevent loss to follow-up and to turn EMZL’s favorable biology into truly favorable long-term outcomes.

## Data Availability

Any inquiries regarding supporting data availability for this case report should be directed to the corresponding author.
